# Cancer: A Tale of Aberrant PRR Response

**DOI:** 10.3389/fimmu.2014.00161

**Published:** 2014-04-09

**Authors:** Raunaq Singh Nagi, Ashish Shekhar Bhat, Himanshu Kumar

**Affiliations:** ^1^Laboratory of Immunology, Department of Biological Sciences, Indian Institute of Science Education and Research, Bhopal, India; ^2^Laboratory of Host Defense, WPI, Immunology Frontier Research Center, Osaka University, Osaka, Japan

**Keywords:** cancer, pattern recognition receptors, innate immunity, inflammation, regulation

## Introduction

Cancer is a disease of complex etiology and multistep progression, manipulating the regular routes to homeostasis. Any deviation from homeostasis alerts the innate immune system and provokes inflammation. Inflammation is generated by the signaling cascades launched by the pattern recognition receptors (PRRs), the germ-line encoded molecules dedicated to sense pathogen, or danger-associated molecular patterns (PAMPs or DAMPs) in case of pathogen/foreign matter invasion and intrinsic disturbances, respectively (1–3). Through inflammation, PRRs eliminate stress signals and re-establish homeostasis in the body, via drawing the required cellular machinery to the inflammatory sites. However, the same lympho-reticular infiltrate has been linked with incidence of cancer at the site of chronic inflammation, since 1863, by Rudolf Virchow (4). From 1990s vast amount of literature has accumulated associating soluble and cellular factors of innate immune system with prevalence and progression of cancer. Furthermore, in the past decade, several pathogens have been linked with cancer as well [Ref. (5, 6) and references therein].

Fascinatingly, it is remarkable how the tightly regulated sensory system for stress removal and maintenance of homeostasis functions anomalously and promotes occurrence and progression of cancers (7–9).

## PRR-Mediated Responses and Cancer Progression

All PRR-dependent pathways activate a particular set of transcription factors to generate appropriate responses. The same factors govern cellular proliferation, apoptosis, tissue remodeling, or angiogenesis, and exhibit a perturbed activity during cancer. One such key protein is nuclear factor κB (NFκB); up-regulation of which leads to production of pro-inflammatory cytokines. Additionally, it induces anti-apoptotic proteins like Bcl2 or inhibitors of apoptotic proteins (IAPs) and angiogenic proteins, such as angiopoietin or vascular endothelial growth factor (VEGF). NFκB also induces nitrous oxide synthase-2 (NOS-2), thus producing nitrous oxide (NO) in the immune cells, which along with reactive oxygen species (ROS) eradicates infected cells by lipid per-oxidation and DNA damage (10–14). Conversely, genomic instability and free radicals thus produced act as DAMPs, leading to sensitization of neighboring PRRs and further immune activation, for instance, the DNA fragments released can activate local DNA sensors, resulting in production of Type I IFN by DAI-TBK1, and activate KRAS pathway of cellular proliferation via TBK1-Sec5 complex, which leads to further activation of NFκB and production of anti-apoptotic proteins (15). That is, detouring regular anti-cancer pathway toward proliferation. Also, RONS induce DNA methylases, which lead to methylation and silencing of tumor suppressor and DNA damage repair genes (2, 16–18).

Another pathway crucial in immunity and cancer is the Janus kinases (JAK)-signal transducers and activators of transcription (STAT) pathway. Triggered primarily by interferons and some other mediators, this pathway stimulates various proliferative genes, such as IL-6-mediated induction of myc and CyclinD1/D2 through JAK; also TNFα-mediated up-regulation of STAT-3 leading to activation of Ras-mitogen activated protein kinase (MAPK) pathway, which leads to the expression of transcription factor activating protein (AP)-1, and epidermal growth factor (EGFs) along with eukaryotic initiation factor (eIF)-4. AP-1 couples with NFκB, inducing matrix metalloproteinase (MMP)-9, a protein involved in tissue remodeling required during angiogenesis (19). Thus, the pro-inflammatory signal culminates in the production of proteins aiding tumor survival, proliferation, and development of tumor-associated vasculature (18).

Furthermore, NFκB is also involved in the expression of NLRP3, which assembles with apoptosis-associated speck-like protein containing a CARD (ASC) caspase-1 to form multi-protein complexes, the inflammasomes, and responds to DAMPs, especially nucleotides released from damaged or necrotic tissue (due to cytotoxicity of free radicals) (20). Likewise, absent in myeloma (AIM)-2 inflammasomes also organize in response to the formation of DNA adducts (DNA and cytosolic protein HMGB-1) from the dying tissue (21). These assemblies lead to activation of IL-1β–IL-1βR pair; a system found commonly over-activated in many cancers (2, 22). Additionally, NFκB also generates cyclo-oxygenase-2 (COX-2) enzyme, which converts arachidonic acid into prostaglandin-E2 (PGE-2), one of the dual (pro-inflammatory and/or anti-inflammatory) mediators of immune response. PGE-2 enhances T-cell activation and represses B-cell activity (23, 24). Another common enzyme, activation-induced deaminase (AID), also induced by NFκB, involved in somatic hypermutation and class switch recombination in B-cells, causes genome instability and releases additional DAMPs into the microenvironment (25). Thus, the immune mediators produced for protection can divert inflammation toward pro-tumor facet (26).

A set of pro-inflammatory cytokines consisting of TNF-α and IL-1 and 6 is essentially tumor directing. TNF-α promotes tumor initiation and DNA damage. It also up-regulates hypoxia-inducible factor (HIF)-1α (attributed to the increasingly low oxygen levels due to multiplying cells) aiding in angiogenesis (27). IL-1β aids in tumor invasiveness and adhesion required during metastasis to new sites. IL-1α, the membrane bound form, induces IL-1 expression, associated with tissue damage, compensatory cell proliferation, and activation of JAK-STAT pathway, as seen in hepatocellular carcinomas and colitis-associated cancers (22, 28).

Cigarette smoking has long been associated with incidence of cancer. Cigarette smoke contains numerous compounds with known cytotoxicity, mutagenicity, and carcinogenicity, most of which are particulate. Stable ROS present in the smoke damage DNA and cause lipid per-oxidation, sensitizing the PRRs present from the buccal cavity to lungs leading to increased IL-8 and TNF-α (11). In addition, both, NFκB and AP-1 are up-regulated exaggerating the pro-inflammatory signal, at the same time homeostatic activity of both is reduced, compensating normal immune response. Such a response coupled with prolonged exposure can spontaneously lead to cellular transformations and their expansion (29, 30).

## Role of Cellular Components of Innate Immunity in Cancer Progression

Specialized cells of the immune system are equipped with PRRs, and are responsible for clearance of diseased/damaged cells. Pro-inflammatory cytokines draw these cells toward the inflammatory site and direct them for removal of pathogens, particulates, or immune debris. These populations recede as the signal resolves. Since Virchow proposed their role at the site of chronic inflammation and cancer, a number of cellular populations and their effector responses have been ascertained for the same. In cases of prolonged exposure to PAMPs/DAMPs, infiltrating cells fail to withdraw and differentiate into M2 macrophages, identified as tumor-associated macrophages (TAMs), an integral population programed for tissue remodeling and tumor progression. Upon activation of their PRRs, TAMs promote various properties of cancer by releasing a range of inflammatory and angiogenic bio-chemicals. These cells stimulate proliferation of stromal tissue and macrophages by growth factors such as platelet-derived growth factor (PDGF) and colony stimulating factor (CSF)-1 respectively (31). Moreover, they organize a route to metastasis by digesting the substratum, basal lamina and release inactive growth factors via, MMPs. In addition, they assist in cellular movements by releasing cell adhesion molecules such as intercellular adhesion molecule (ICAM)-1 (32–34).

Another such population, the NK cells meant to carry out cytotoxic clearance of all the cells which do not express human leukocyte antigen (HLA) A/B/C and thus fail to activate the membrane-expressed inhibitory receptors (NKp30/44/46). NK cells are also responsible for killing any cell, irrespective of HLA tag, which presents them with stress/abnormality/tumor-associated antigens, via, activating receptor (NKG2D). In addition, they also participate in antibody-dependent cell-mediated cytotoxicity (ADCC), on cells tagged through FCRγIII (35, 36). A number of cytokines activate NK cells and turn them into lymphokine-activated killers, causing them to display their killing property at the site of recruitment (37). Tumor cells escape NK cells by blocking the activating receptor. Also, even if recruited, NK cells can only bring about killing of the outer cells in a solid tumor (38, 39). Furthermore, a reduction in NK cell cytotoxic function as well as NK cell dependent tumor surveillance is evident as a tumor directing effect of cigarette smoke (29, 30).

Another newly characterized population of cells called myeloid-derived suppressor cells (MDSCs) are recruited by inflammatory mediators, which inhibit the anti-tumor responses and release pro-tumor molecules, like, NOS-2 and TGF-β. Arginase-1 and indolamine-2,3-dioxygenase produced by MDSCs are involved in silencing the anti-tumor immunity by reducing T_h_1 activization (40, 41).

Soluble and cellular factors work in union. Cellular proliferation at an enhanced rate at tumor sites gives rise to hypoxia, low concentration of oxygen that stabilizes HIF-1α, which activates NFκB to secrete an angiogenic protein, VEGF and also induces expression of IL-12 and TNF-α. These mediators collectively induce STAT-3 production that up-regulates PGE-2 which further recruits NK cells and their cytotoxic function releases DAMPs in vicinity attracting TAMs which aid in shaping neo-vasculature and creating area for growing cell mass. Deregulation of T_h_1 responses by PGE-2 and MDSC/TAMs creates an imbalance (42–44). HIF-1α which causes glycolytic environment, such conditions may tip the balance into a state when IL-12 production is replaced by IL-23, that is, a shift from anti-tumor to pro-tumor (45, 46). In this manner, the PRR triggered pathways to eliminate PAMPs/DAMPs, are rerouted in a manner that leads to exaggeration of the initial signal causing chronic inflammation; moreover eliciting other pathways concluding in tumor growth and metastasis (1, 47, 48).

## Regulatory Mechanisms and Progression of Cancer

To maintain homeostasis in the body, all cellular processes, including PRR generated immune responses are regulated by various mechanisms. This control is exercised at various levels. At transcriptional level certain cytokines can inhibit transcription factors, such as IL-4/13 which hinder NFκB. Alternatively; some cytokines directly inhibit other cytokines at protein level, such as inhibition of TNF-α by IL-10. At post-transcriptional level microRNAs play a crucial role by either resolving inflammation or potentiating pro-tumor effects of cytokines (18). For instance, TNF-α/IL-1β induced mir-146a inhibits IRAK/TRAF6, that is, the downstream signaling of TLR pathway, thus resolving inflammation (23). In contrast IL-6 induced mir-21 targets tumor suppressor genes, such as phosphatase and tensin homolog (PTEN), programed cell death (PDCD)-4, and others, thereby hampering the anti-tumor effects (49–52). Another microRNA found commonly up-regulated in cancer, miR-155, induces NOS-2 and inhibits apoptosis by down-regulating TP53INP1 (a downstream molecule of p53 signaling) (53). Thus, a slight imbalance in the pro-inflammatory and anti-inflammatory signals can shift the equilibrium toward oncogenic transformations (18).

**Figure 1:**
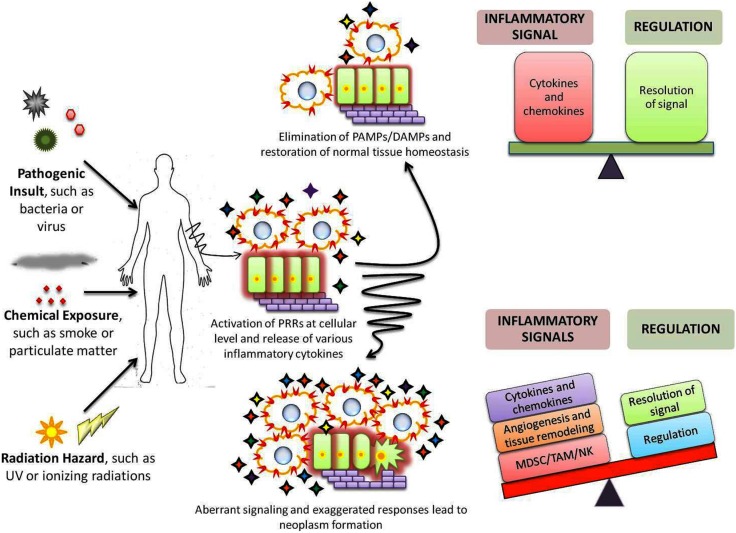
**Role of PRRs in maintenance of homeostasis and cancer development**. Exposure to any of the various stress stimuli leads to activation of immune system through PRRs (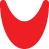) which results in expansion of a variety of cellular populations and production of numerous cytokines (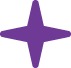). A precise sensitive balance at the inflammatory and regulatory front eliminates and reinstates homeostasis within the host. However, under certain circumstances, the precise balance is altered due to aberrant/exaggerated inflammation or failure of regulatory mechanisms to restrict them. Such imbalance results in accumulation of cells and chemicals that deregulate the proliferative pathways; induce angiogenesis and damage the extracellular matrix, thus aiding in origin and/or progression of cancers.

Pattern recognition receptor elicited signaling cascades induce synthesis of several zinc-finger proteins that help in regulating the inflammatory signals. For example, ZC3H12a/c and Zfp36 proteins degrade the cellular mRNA coding for pro-inflammatory cytokines; while ZAP proteins directly degrade the viral RNA, to be precise, the PAMP itself (54, 55). Thus, these proteins degrade the PAMPs or signal generated by them to curb inflammation. In addition, certain PRRs themselves restrain the downstream signaling such as inhibition of IPS-1, adaptor molecule of RLR, by NLRX-1. Mutations within these genes or their regulatory elements or imbalance at cellular level can skew the balance toward tumorigenesis (6, 55) (Figure 1).

## New Facet in the Field

A relatively new but noteworthy field in analyzing cancer biology is polymorphisms. Single nucleotide polymorphisms (SNPs) are single base changes in the DNA, which may produce totally drastic effects on the structure and function of the encoded molecules. PRRs and the associated machinery, such as the adaptors or receptors are also susceptible to such polymorphisms and many such SNPs have been reported. SNPs could promote the anti-tumor effect if they prevent the PRR cascade from commencing, or support the pro-tumor effect, if they cause spontaneous induction of signaling without any stimulus. The cytoplasmic domains of the PRRs and cytokine receptors are of prime importance in this context as they form the docking site for progression of inflammatory response. Many polymorphisms have been identified to be associated with cancers of various origins. Mostly CLRs and RLRs have been associated with sensing PAMPs of oncogenic origin, and polymorphisms in their genes have been correlated with cancers of mesoderm, endoderm, and also ectoderm origin. Also several genes and mutations have been correlated with cigarette smoke in association with cancers (56). Still, a rather comprehensive endeavor would be required to establish the integral role of these SNPs at the molecular level to outline their part in incidences and progression of cancers [Ref. (6) and references therein, Ref. (56)].

## Conclusion

Recognition of pathogen/stress is one of the essential processes of the host. Tumor cells are in fact, abnormal cells which are steadily eliminated through PRR-mediated pathways. However, hyper or anomalous behavior of same pathways can divert the protective route toward malignancy, by contributing to abnormal proliferation, angiogenesis, or modifying tissue architecture. The origin of anomalous behavior maybe external and internal, such as pathogen/foreign insults tissue damage/necrosis or mutations and polymorphisms in vital signaling components, respectively. Unfolding the root of these irregularities and malfunctions shall help in better understanding of the disease and thus, create new and personalized prospects for treatment.

## Conflict of Interest Statement

The authors declare that the research was conducted in the absence of any commercial or financial relationships that could be construed as a potential conflict of interest.
